# Opening a “Can of Worms” to Explore the Public's Hopes and Fears About Health Care Data Sharing: Qualitative Study

**DOI:** 10.2196/22744

**Published:** 2021-02-22

**Authors:** Olivia Lounsbury, Lily Roberts, Jonathan R Goodman, Philippa Batey, Lenny Naar, Kelsey M Flott, Anna Lawrence-Jones, Saira Ghafur, Ara Darzi, Ana Luisa Neves

**Affiliations:** 1 Patient Safety Translational Research Centre Institute of Global Health Innovation London United Kingdom; 2 Patient Safety Movement Foundation Irvine, CA United States; 3 The Helix Centre Institute of Global Health Innovation London United Kingdom; 4 Centre for Health Policy Institute of Global Health Innovation Imperial College London London United Kingdom; 5 Center for Health Technology and Services Research / Department of Community Medicine, Health Information and Decision Faculty of Medicine University of Porto Porto Portugal

**Keywords:** electronic health records, patient participation, data sharing, patient safety, data security

## Abstract

**Background:**

Evidence suggests that health care data sharing may strengthen care coordination, improve quality and safety, and reduce costs. However, to achieve efficient and meaningful adoption of health care data-sharing initiatives, it is necessary to engage all stakeholders, from health care professionals to patients. Although previous work has assessed health care professionals’ perceptions of data sharing, perspectives of the general public and particularly of seldom heard groups have yet to be fully assessed.

**Objective:**

This study aims to explore the views of the public, particularly their hopes and concerns, around health care data sharing.

**Methods:**

An original, immersive public engagement interactive experience was developed—*The Can of Worms* installation—in which participants were prompted to reflect about data sharing through listening to individual stories around health care data sharing. A multidisciplinary team with expertise in research, public involvement, and human-centered design developed this concept. The installation took place in three separate events between November 2018 and November 2019. A combination of convenience and snowball sampling was used in this study. Participants were asked to fill self-administered feedback cards and to describe their hopes and fears about the meaningful use of data in health care. The transcripts were compiled verbatim and systematically reviewed by four independent reviewers using the thematic analysis method to identify emerging themes.

**Results:**

Our approach exemplifies the potential of using interdisciplinary expertise in research, public involvement, and human-centered design to tell stories, collect perspectives, and spark conversations around complex topics in participatory digital medicine. A total of 352 qualitative feedback cards were collected, each reflecting participants’ *hopes* and *fears* for health care data sharing. Thematic analyses identified six themes under *hopes*: enablement of personal access and ownership, increased interoperability and collaboration, generation of evidence for better and safer care, improved timeliness and efficiency, delivery of more personalized care, and equality. The five main *fears* identified included inadequate security and exploitation, data inaccuracy, distrust, discrimination and inequality, and less patient-centered care.

**Conclusions:**

This study sheds new light on the main hopes and fears of the public regarding health care data sharing. Importantly, our results highlight novel concerns from the public, particularly in terms of the impact on health disparities, both at international and local levels, and on delivering patient-centered care. Incorporating the knowledge generated and focusing on co-designing solutions to tackle these concerns is critical to engage the public as active contributors and to fully leverage the potential of health care data use.

## Introduction

### Background

With the advent of the digital age, health care professionals have witnessed significant advancements in innovation and research. The increasing adoption of digital technologies and electronic health records (EHRs) has expanded the capacity for interoperability and data sharing, both for primary uses (ie, direct care) and secondary uses (ie, research, public health, health policy). Evidence suggests that health care data sharing may strengthen care coordination, improve quality and safety, and reduce costs [1]. However, the growing complexity of systems, stakeholders, and unbounded ecosystems, unsuccessful data sharing initiatives (eg, the care.data program in the United Kingdom), and recent cybersecurity incidents (eg, the WannaCry attack) and evolving regulations (eg, General Data Protection Regulation) [2] have contributed to the increasing lack of clarity and trust by the public [3]. Therefore, data sharing is becoming an increasingly controversial subject, with many researchers and patients reporting concerns about how and why health care data are shared [4]. Previous evidence highlights that the most common concern is patient privacy [5,6]; even when data are anonymized, there remains a risk that by using only a few data points, patients can be reidentified by their own health information [7]. Previous studies also highlight that public support is generally higher when data are used for the *greater good*, but the acceptance rates fall steeply when data are shared for use by commercial companies [8].

In recent years, several health care data sharing initiatives have been implemented globally. HealthData Research UK, as part of the Industrial Strategy Challenge Fund, has launched 7 new data hubs as part of a 4-year program to create a UK-wide system for the secure and safe use of large-scale health data [9]. In the United States, the Institute for Healthcare Improvement’s Triple Aim Initiative uses geographic health data to comprehensively understand population health by location, with the aim of improving the experience of care, health of populations, and cost-effectiveness [10]. Innovative engagement programs, as opposed to traditional banners, posters, and advertisements, are especially promising for promoting an informed choice [11]. Over the past several years, social media campaigns and even live theatrical performances have been used to improve the understanding of data sharing practices [12,13]. However, to achieve efficient and meaningful adoption of health care data sharing initiatives, it is necessary to engage all stakeholders, including policy makers, health care professionals, researchers, patients, and the public. A broad understanding of their views, hopes, and concerns about data sharing is crucial to frame the breadth of perspectives, increase adoption, support progress, and enhance equity of care delivery.

Previous research has largely focused on health care provider perspectives and found that providers hope data sharing can have a positive impact by tailoring and improving care delivery [14-17]. Previous research addressing health care professionals’ views identified several benefits of data sharing: improved population health, ease of access, and reduced costs [17-19]. However, talking about data sharing is like opening a metaphorical can of worms: when the subject is brought up, many concerns also emerge, including issues around patients’ willingness to share, trust, privacy, transparency, confidentiality, and security [20].

If data are anonymized, many patients are comfortable with sharing for the improvement of health services and care [21], with as many as 88% of patients trusting the United Kingdom National Health Service (NHS) to store data safely and use it for ethical, research-oriented reasons [22]. Conversely, a recent survey showed that only one-tenth of people would share data willingly with a tech company [23]. Transparency seems to be a critical factor for patients’ willingness to share their data, as the more transparent the organizations are with the public about the use of health care data, including, but not limited to, who has access, the rights to access, and the safeguarding processes in place, the greater the public support for data sharing initiatives [24]. However, a deeper exploration of the factors that contribute to the willingness to harness health care data from the general public perspective is still lacking.

In addition, policy and structural changes are necessary to promote a culture of safety and transparency in organizations across the continuum of care [25]. These changes must be standardized and embedded at every level of care, including primary care, secondary care, and community services at local and national levels, to ensure integrity and alignment, and should be guided by international quality standards. Understanding and integrating the public’s hopes and concerns into these policy and structural changes is fundamental to ensure the development of patient-centered data sharing policies.

### Objectives

This qualitative study aims to explore public views, particularly their hopes and concerns, around health care data sharing.

## Methods

### Overview

We developed an original, immersive public engagement interactive experience—*The Can of Worms* installation—with which we aimed to challenge the conventions of how members of the public receive and give information, using interdisciplinary expertise in research, public involvement and human-centered design, to tell stories, collect perspectives, and spark conversation around complex topics.

To meet the aims and objectives of this study, a qualitative descriptive approach was adopted. Qualitative methods were chosen because of their ability to capture descriptive data on individual perceptions, attitudes, and behaviors [26]. A multidisciplinary team including medical doctors (AN, SG, and AD), health service researchers (JG, LR, KF, and OL), designers (PB and LN), and a patient and public involvement specialist (AJ) with previous experience in qualitative research designed the *Can of Worms* concept and performed this study. Members of the public were involved in recruitment, developing the *Can of Worms* concept and reviewing materials.

#### Recruitment

A combination of convenience and snowballing sampling was used, and no other exclusion criteria (apart from age) were adopted, to optimize the diversity of the sample. Members of the public (<18 years) were invited to participate through a combination of recruitment approaches, including public advertising through posters, distribution of flyers, partners’ networks, social media, and word of mouth. The research team had no established relationships with the participants before the event. Free tea and coffee were offered to the participants; however, no financial incentives were provided for participation.

#### Concept Development

The *Can of Worms* metaphor was used as a novel way of bringing interest to the subject matter. The exhibition design employed a multidisciplinary team of designers, public members, and researchers to craft a user-centered experience for participants. The team prioritized flexibility, inclusivity, and engagement, principles that guided the design process.

**Figure 1:**
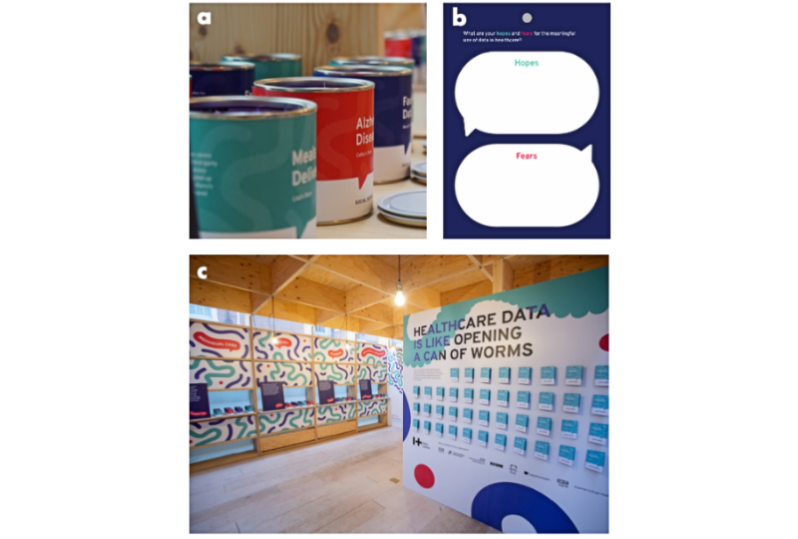
Materials used in the *Can of Worms* events. (a) Cans containing audio stories focusing on a particular aspect of healthcare data sharing. (b) Blank response card used to collect participants' hopes and concerns towards data sharing. (c) Overview of the *Can of Worms* public engagement immersive installation.

People were invited to explore a free (no admission fee) and immersive installation (ie, in which the space was designed to impact the experience for visitors, as described below). Participants were prompted to reflect on the subject of health care data sharing through storytelling and conversation. Visitors were given a *can opener* and an information leaflet and were encouraged to open cans and listen to stories about health care data sharing, stored in a recorder within the can (Figure 1). Each of the 28 fictional stories fit under 1 of 7 categories: diagnosis, individual care, planning, policy, social services, treatment and prevention, and understanding disease. Topics included international data sharing among patients with chronic diseases, genomic analysis, and data-powered predictive algorithms. The fictional stories were developed by researchers based on anecdotal evidence of risks and benefits of data sharing to represent a balanced view. They were further reviewed by clinicians and lay partners to ensure that they were relevant, factual, and realistic, not harrowing, and in plain English. Stories were recorded by a variety of actors and public members to ensure that they were engaging and relatable. Large text-printed versions were also available for anyone with specific access needs. Examples of stories can be found in Multimedia Appendix 1 [27] and a full list of all stories used within the *Can of Worms* installations can be found at the project website [27].

### Data Collection

Once participants finished listening to some of the recorded stories, they were asked to provide the following information on a card: age bands, hopes, and fears about the meaningful use of data in health care (Figure 1).

The first exhibition took place at the Helix Pop-up within St. Mary’s Hospital in Paddington, London, in November 2018, which meant that the members of the public entering the space were mostly patients, family members, medical students, and passersby near the train station. To encourage the participation of people from seldom heard groups (eg, those from deprived backgrounds and minority ethnic groups), a free bus service was organized from White City and Woodlane to the event. The second was held over a weekend at The Great Exhibition Road Festival, South Kensington, in June 2019—*Can of Worms* was one of many exhibits at an event attracting 20,000 members of the general public, as well as students and staff. The third took place on one day at the NHS Digital Academy Residential in Tower Hill in November 2019—this event was open to participants on an academic program for digital health leaders.

Participants’ responses were compiled verbatim and were not returned to the participants for comments and/or corrections. As anonymous, self-administered cards were used, only the participant was present when registering data. Participants had minimal knowledge of the research team; thus, the potential for bias and assumptions was kept to a minimum. No repeat interviews were conducted.

### Data Analysis

The transcripts were compiled verbatim and systematically reviewed by four independent reviewers using the thematic analysis method to identify emerging themes. The themes were supported by participants’ quotations. The Consolidated Criteria for Reporting Qualitative Research were used to ensure that the study met the recommended standards for qualitative data reporting [28].

### Ethics

The project was reviewed by the Health Research Authority (HRA) Public Involvement Team HRA. Additional HRA approval by the NHS Research Ethics Committee review was not deemed necessary [29].

## Results

### Participant Characteristics

A total of 352 participants filled the response card (iteration 1 [Helix Centre]: n=175; iteration 2 [Exhibition Road Festival]: n=142; iteration 3 [NHS Digital Academy]: n=35). In iteration 1, the most frequent age band was 25 to 34 years (51/175, 29.1%). In iteration 2, the most frequent age band was below 18 years (55/142, 38.7%), and in iteration 3, the most frequent age band was 45 to 54 years (27/35, 77%).

The themes presented are listed in no particular order, and in line with the qualitative approach, no one theme is presented as more important than the other. 

#### Hopes

The level of content and detail varied greatly between cards. Thematic analysis of the patients’ narratives revealed six emerging overall hopes: (1) enablement of personal access and ownership, (2) increased interoperability and collaboration, (3) generation of evidence for better and safer care, (4) improved timeliness and efficiency, (5) delivery of more personalized care, and (6) equality (Textbox 1).

**Hopes regarding health care data sharing.**

**Theme 1: personal access and ownership**

- “Patients will hold their data and will share what they want with who they want” (ID 155)
- “Data will be there for every patient when they need it and it will follow them” (ID 144)
- “Having your medical data available on your mobile/smart watch could save many lives in an emergency in the future” (ID 65)

**Theme 2: interoperability and collaboration**

- “That we treat all data collected across the NHS as one, rich resource. At the moment, there are hundreds of data controls in the NHS and sharing is difficult” (ID 160)
- “For seamless, secure sharing of data between patients, GPs, A&E… so that patients can get the best care possible” (ID 37)
- “As a doctor, I find it difficult to provide the best care for my patients without full access to their past medical history, previous imaging and tests, and up to date medication lists. Data sharing between trusts and GPs” (ID 20)
- “The data can be shared to ensure that there is access to information, especially out of normal hours, so that clinicians can always make informed decisions” (ID 149)
- “That all my healthcare data is available to any medical professional I see! To save me time and inaccuracy” (ID 184)
- “Data sharing means I don’t have to tell my story again and again” (ID 185)
- “A more open and collaborative approach to healthcare across the globe” (ID 183)

**Theme 3: evidence for better and safer care**

- “Understandings that will help the whole healthcare sector from big data analysis” (ID 13)
- “Analysing huge amounts of data could help to find out what causes different diseases (...)” (ID 33)
- “I hope keeping and sharing patients’ data will lead to more efficient and accurate diagnoses avoiding human error and be able to draw on a bigger database that slips the human mind” (ID 186)
- “Data can be enhanced to improve outcomes for patients [via] better and quicker diagnosis” (ID 187)
- “Provides an evidence base for identifying effective treatments” (ID 143)
- “Analysing huge amounts of data could help to (...) see and predict how epidemic illnesses are spreading” (ID 33)
- “Less likely for mistakes to happen” (ID 55)
- “Investment and development of technology to achieve parity with industry with human factors and evidence-based design and implementation” (ID 152)

**Theme 4: timeliness and efficiency**

- “That data gets where it needs to at the time it needs to. Patient care is supported and improved by timely access to the right information” (ID 165)
- “(...) an ecosystem that provides pace and a streamlined service – think how much quicker we could help people” (ID 182)
- “Diseases can be detected early on” (ID 73)
- “Broad sharing and easy access of data to help in a quicker understanding of a healthcare issue and [potential cure]” (ID 4)
- “It creates a clear picture of who I am – so I can be helped better and minimize waste in the health service” (ID 180)

**Theme 5: personalized care**

- “That it will bring a smarter, more cohesive, personalized care” (ID 179)
- “Reduction in anxiety to have to tell your story [repeatedly]” (ID 157)
- “Improving preventive behavior through personalized interventions” (ID 148)

**Theme 6: equality**

- ”That greater ownership by patients of their data will encourage conversations of equality between them and their healthcare providers” (ID 181)
- “The patient data can be used and shared more effectively, for example, [in the care of] transgender patients, the proper pronouns can be used” (ID 9)
- “Data is a very powerful way to tackle inequalities and improve the level of care” (ID 87)

#### Personal Access and Ownership

Participants hoped that health care data sharing will enable patients access to their own medical records, improving their sense of ownership and involvement in their health and care. Participants also highlighted that improved accessibility of health care data by the patient could prove pivotal in improving safety in medical emergency situations.

#### Interoperability and Collaboration

The opportunity for enhanced interdisciplinary engagement across the health care field was another hope identified in this study. Participants from each iteration hoped for a more united health care system, ease of collaborative care, and fortified capacity for health care providers to make informed decisions anywhere at any time. Participants hoped for the treatment of data collected as a rich resource instead of the current, fragmented state, which compromises quality and safety of care.

#### Evidence for Better and Safer Care

Participants also highlighted that data sharing in health care can contribute to providing better and safer care. A significant number of participants from all iterations hoped that data sharing could pave the way for analytical and data mining approaches to improve clinical knowledge in several aspects, including understanding etiology and improving diagnosis and effectiveness of treatment. Some participants also acknowledged that health care data sharing can help understand and monitor the epidemiological nature of certain diseases.

#### Timeliness and Efficiency

Participants expressed how they hoped that health care might become more efficient as a result of data sharing, as data may be accessed anytime, anywhere. Some saw this from the angle that waste could be minimized and more patients helped if data were shared more widely.

#### Personalized Care

Another theme emerging from the responses was the hope that health care data sharing would lead to more personalized care. Participants highlighted that it could reduce the anxiety produced by having to tell their story repeatedly due to a lack of integrated health records. Data sharing could also result in a more cohesive health record that could allow care to be tailored to individual needs.

#### Equality

Participants expressed their hope that data sharing would allow patients to be treated equally, regardless of their backgrounds, predispositions, access to public care, or financial means for private care.

### Fears

Regarding the main fears, the thematic analysis of the public’s narratives revealed five emerging themes: (1) inadequate security and exploitation, (2) data inaccuracy, (3) distrust, (4) discrimination and inequality, and (5) less patient-centered care (Textbox 2).

**Fears regarding health care data sharing.**

**Theme 1: inadequate security and exploitation**

- “Issues around privacy of data” (ID 194)
- “This data being used by companies to discriminate and make [profit]” (ID 37)
- “That conclusions are made without proper examination of the data [and] that the data is used for nefarious purposes” (ID 143)
- “That private companies could use this data purely for targeting the public health service, thereby driving up the costs” (ID 163)

**Theme 2: data inaccuracy**

- “Incorrect data” (ID 161)
- “Errors that could corrupt the data” (ID 158)

**Theme 3: distrust**

- “Horror stories delay inevitable progress” (ID 153)
- “That just one rotten egg will set us back years and we miss out on all the progress that could be made” (ID 196)

**Theme 4: discrimination and inequity**

- “Could widen inequalities for countries that can’t afford these technologies” (ID 189)
- “Introduces bias and fails to support a patient-centered approach in a mental health and community setting” (ID 147)
- “Bias gets perpetuated” (ID 150)
- “That if my serious and ongoing medical conditions get out that it would limit or otherwise negatively impact my career pathway and job options” (ID 178)

**Theme 5: less patient-centered care**

- “The ‘person’ is being lost and replaced with numbers” (ID 195)
- ”We become too reliant on data and forget about the individual patient” (ID 169)
- “We no longer have conversations with healthcare professionals... many may be replaced by exchange of statistics” (ID 30)
- “Artificial intelligence is seen as a quick and cheap fix and patients get substandard care” (ID 159)

#### Inadequate Security and Exploitation

Participants conveyed their concerns that health care data sharing could be associated with a lack of privacy and security and would therefore be potentially used for nefarious purposes. Specifically, individuals feared the potential for private companies (ie, pharmaceutical companies) to leverage the data for profit at the expense of the public.

#### Data Inaccuracy

Data accuracy was also a concern, with some participants expressing worries about incorrect data in their records or computing mistakes that may corrupt their data. Participants expressed worry about the accuracy of communication between clinicians and were concerned that the overreliance on data might further compromise communication.

#### Distrust

Participants expressed a wide variety of perspectives that shared an overall feeling of distrust and apprehension about the potential for sustained and adequate adoption. Some were wary of the impact of previous negative experiences and how they may impact future initiatives of health care data sharing.

#### Discrimination and Inequity

Participants expressed concerns that increased health care data sharing would only be possible in better-connected regions, and this could widen the gap between these regions and those that do not have the resources to implement such technologies. On an individual level, participants writing from a patient’s perspective were concerned that, if shared widely, certain details of their personal data may introduce or perpetuate biases.

#### Less Patient-Centered Care

Some participants were concerned about a negative impact on the delivery of care that is respectful of, and responsive to, individual patient needs, preferences, and values. Fears were expressed around health care, becoming too focused on data, with a negative impact on the patient-doctor relationship, communication, and quality of care received. Some were concerned that a strong focus on data and artificial intelligence could have a negative impact on patient centeredness.

## Discussion

### Principal Findings

The six main *hopes* that participants had for health care data sharing concerned (1) enablement of personal access and ownership, (2) increased interoperability and collaboration, (3) generation of evidence for better and safer care, (4) improved timeliness and efficiency, (5) delivery of more personalized care, and (6) equality. The five main *fears* that participants expressed in relation to health care data sharing were (1) inadequate security and exploitation, (2) data inaccuracy, (3) distrust, (4) discrimination and inequality, and (5) less patient-centered care.

### Findings as Compared With Previous Studies

In this study, participants highlighted personal access and ownership over their health records as a key *hope* for data sharing. This sentiment has been expressed in previous studies; a public engagement exercise by the Wellcome Trust (2010) found that 92% of adults and 97% of young people surveyed supported patient access to their own health records [30]. Furthermore, a recent meta-analysis has shown that providing patients with access to their own records can improve several aspects of quality of care, particularly improving health outcomes and patient safety [31]. The growing body of evidence supporting policies that support data sharing with patients also raises important questions about equity and whether these interventions may exacerbate health inequities by improving outcomes for patients with access to their health care data, while further excluding those with low health literacy or poor access to digital technologies [32,33].

Interoperability and collaboration also emerged as key hopes for data sharing in this study and were described from various perspectives as allowing clinicians to make more informed decisions and avoiding the need for patients to repeatedly narrate their clinical information. Interoperability between systems and care settings is recognized as a key aspiration to achieve the full potential of data sharing [34], and it is necessary to engage stakeholders involved (patients, health care professionals, policy makers, and technical companies). Several aspects need to be considered, including the adoption of international standards [35], improved education and awareness of obstacles, and minimizing privacy and cybersecurity issues [34].

The hope that data sharing would provide evidence for better and safer care is in line with the findings of previous studies. A study by O’Brien et al [36] found that 94% of patients surveyed “thought data sharing would help their doctor to make better decisions about their health”. In the last decade, the United Kingdom has witnessed a surge in the secondary use of health care data that has generated population-based evidence to inform the delivery of better care, particularly in the mental health space [37] and prescription patterns [38]. Similarly, in the United States, pilot studies have started predicting readmissions and estimating the risk of complications in newborns [37].

Improved timeliness and efficiency were also identified as key hopes in this study, whether it was for patient benefit (ie, early diagnosis and treatment, improvement in diagnosis) or for improved health care efficiency (ie, allowing care to be delivered faster and to more people). Our findings are in line with previous studies surveying patient perspectives, in which helping make new therapies available faster was one of the 2 most important perceived benefits of data sharing [36].

Importantly, participants hoped that data sharing might contribute to making health care more inclusive and increase transparency around demographic information, particularly gender preference, where the use of proper pronouns is of utmost importance to an individual’s identity. Previous research suggests that allowing patients to self-label their gender in their EHRs and specifying their preferred names and pronouns could improve their health care experience [39].

The most common *fear* noted by participants was inadequate security and the exploitation of health data. Following the WannaCry attack of 2017, public confidence in the NHS to handle data has been negatively affected [40]. A web-based patient survey in the United Kingdom found that the most important data sharing risk identified by participants was health data being *stolen by hackers* and that in general they would be more comfortable if they were able to learn how their data were protected [36]. These findings mirror the hopes of health care professionals who acknowledged some of these concerns in previous research [41]. Closely linked with fears regarding inadequate security and exploitation of patient data is the idea that distrust can delay progress and hinder the realization of benefits from data sharing. In line with these findings, previous studies showed that the public opposed data sharing when there were financial gains or profits or the possibility of sharing their health care data with private or commercial companies [42].

Errors that resulted in data inaccuracies were feared by the study participants. Previous studies have also found that the public is concerned that errors in records may be difficult to correct and have a negative impact on their care [40]. Previous research highlights that when using data as evidence for better care, it is important that data quality is prioritized, as only with high quality, clean data to feed artificial intelligence algorithms can meaningful insights be extracted [43].

Fears that increased data sharing would give rise to discrimination and inequity of patients were voiced from different contexts. Our participants were concerned that health disparities would widen between more developed countries and those that could not afford these technologies. Others were concerned that some patients may be unfairly prioritized over others, a point of view that is shared with other studies [40]. In one study, those with a lower socioeconomic status expressed more concerns about data sharing and were less likely to consider the benefits that it offers to society [44]. Discrimination, stigmatization, exploitation, or other repercussions are concerns that have been voiced by participants in similar investigations [42].

Some participants expressed that the increased use of computers and artificial intelligence would diminish patient-centered care. The human touch aspect of being treated by a person rather than a computer was valued by members of the public, and there was concern that this would be attenuated significantly. Although digital solutions can improve patients’ safety and efficiency of care, they are not able to replace humanistic skills (ie, compassion, commitment, or empathy) [45].

### Strengths, Limitations, and Future Work

This study has several strengths. We used multiple iterations of data collection, coupled with the triangulation of interpretations between researchers with expertise in qualitative research, clinical research, and cognitive science. Data collection was performed in several settings to capture the perspectives, hopes, and concerns of a diverse study sample. This is crucial to inform an equitable approach to increase data sharing in the future.

This study employed a methodologically rigorous approach, leveraging qualitative methods to capture rich, descriptive data on individual perceptions, attitudes, and behaviors [46,47]. In addition, all data collection and analysis were performed according to the Consolidated Criteria for Reporting Qualitative Studies criteria [48].

On a broader scale, the *Can of Worms* installation is replicable and adaptable for different settings and contexts and can be implemented with relative ease for future installments, both for data collection purposes and to enhance awareness and behavioral change in diverse audiences on this subject.

Despite its strengths, this study has some limitations. The sample size (N=352) was small and was sourced from three locations in England. For this reason, and the fact that data sharing sentiments differ between countries, our results may not be representative of the wider UK population or extrapolated to international settings. The attempt to keep the length of the survey short and encourage those who do not normally engage to participate limited the amount of information collected on participant demographics. As contact information was not collected, it was not possible to send the themes back to the participants for feedback. Future research may benefit from involving patients more actively as part of this process, either by allowing them to provide feedback on the findings, or by providing training so that they can actively contribute to the thematic analyses.

However, the minimal request for participant disclosure of information could serve both as a limitation and a strength of this study, as it could also have increased participation rates and reduced information bias. Finally, the quasi-experimental nature of the study, in an attempt to elicit attitudes, may impact the generalizability of the resulting views, as participants were primed on the stories in the cans. The feedback cards were displayed on a feedback wall in the installation. Some people chose to read these before they wrote their feedback, which could have biased their results but, equally, may have prompted them to have a deeper reflection, including other participants’ perspectives.

Future work should include methodologically robust quantitative studies focusing on how different factors (demographic and social, patient activation, health, and digital literacy) influence both the general public and professional views on data sharing. Future research should also explore the underlying reasons for the public sentiments expressed by collecting additional insights from a range of study participants. Therefore, this study can serve as a first step to unveil areas for future research, from which more actionable conclusions can be drawn. In addition, future work might benefit from international projects assessing data sharing perspectives, as this may help anticipate possible challenges and solutions before future translational implementation of data sharing mechanisms. Finally, future research may consider assessing social media responses to the installations in addition to qualitative responses based on prompts to highlight similarities and contrasting perspectives based on the feedback mechanism.

### Conclusions

In the broader context of sharing health care data, involving the public is critical to create a patient-centric culture in health care systems [49]. This study sheds new light on the main hopes and fears of a sample of the UK public regarding health care data sharing. Importantly, our results highlight novel concerns from the public, particularly regarding the impact of health disparities on delivering patient-centered care. Incorporating the knowledge generated and focusing on co-designing solutions to tackle these concerns are critical to engage the public as active contributors to this decision-making process and to fully leverage the potential of health care data use.

## Appendix

Multimedia Appendix 1 Two examples of stories used in the Can of Worms events. The stories were read by amateur actors, volunteer patient advocates, and researchers. A more detailed overview of the stories is provided in the project website.

Multimedia Appendix 2 COREQ checklist.

## Abbreviations

EHR: electronic health record
HRA: Health Research Authority
NHS: National Health Service
