# Supplementary material for: Opening a “Can of Worms” to Explore the Public's Hopes and Fears About Health Care Data Sharing: Qualitative Study
Source: J Med Internet Res. 2021 Feb 22;23(2):e22744. doi: 10.2196/22744 (PMC7939935; doi:10.2196/22744)
Supplement: Multimedia Appendix 1 [file jmir_v23i2e22744_app1.pdf]

Appendix 1

Two examples of stories we used are given below. We recorded the stories as read by amateur actors, volunteer patient advocates, and researchers at IGHI. A more detailed overview of the stories is provided in the project website (http://www.datacanofworms.com/data-stories).

Faxing Data

Category: Social Services

Narrator: Margot

"I still love to play tennis, but since my knee injury a few years ago it's become a lot harder. A few months ago I hurt it again, and since then the pain has become so bad that it's become difficult to walk.

My doctor recently recommended a physiotherapist who specialises in treating patients with sports-related knee injuries. He's known to tailor the treatment plan to the specific patient, and asked my doctor to fax him my health records.

'Fax?' I asked, when my doctor told me this. 'Like a paper fax? Who uses fax machines anymore?'

'It's still pretty common in the healthcare world, especially in the UK,' she answered. 'In fact, earlier this year I heard the NHS is the world's biggest purchaser of fax machines.'

I was surprised, as I've heard fax machines aren't exactly the most secure way to share information, but if that's how it works and my treatment helps me play tennis again, who cares?"

Diabetes

Category: Understanding Diseases

Narrator: James

"Michelle's had diabetes for a fair few years now. She has type I, which means she can't produce enough insulin to regulate the sugar in her bloodstream. In the past she had to inject himself with insulin throughout the day. It was hard to make sure she got the amount right, it also wasn't very discreet.

Using an insulin pump allows her to treat her diabetes discreetly, which she wears continuously. This reads her glucose levels the pump calculates the right dose of insulin. I also downloaded a phone app for her to monitor her diet throughout the day according to the insulin readings, which the pump monitors. I'm a bit of a worrier so it's good to have that peace of mind.

The technology is great, but sometimes I worry about the app's security. This is really private information, and with all the news about cybersecurity recently, sometimes I worry about keeping Michelle's health information on a third-party app. What if someone stole her information? And how can we be sure the people who designed the app aren't just interested in using Michelle's health data for their own purposes?"
